# A Comprehensive Physiotherapy Rehabilitation for Lower Limb Amputation as a Consequence of Cannabis and Alcohol Dependency: A Case Report

**DOI:** 10.7759/cureus.31700

**Published:** 2022-11-20

**Authors:** Sidra Ahmad Siraj, Chaitanya A Kulkarni, Shubhangi Patil

**Affiliations:** 1 Community Health Sciences, Ravi Nair Physiotherapy College, Datta Meghe Institute of Medical Sciences, Wardha, IND; 2 Community Health Physiotherapy, Ravi Nair Physiotherapy College, Datta Meghe Institute of Medical Sciences, Wardha, IND

**Keywords:** case report, physiotherapy rehabilitation, marijuana, alcohol, amputation

## Abstract

Chronic marijuana and alcohol consumption leads to many forms of physical and physiological deterioration in the human body, mainly affecting the neurological system. A ​​​​65-year-old male patient suffered from stroke with the involvement of middle cerebral artery of the left side. Nine years later, patient presented with gangrene due to a traumatic unhealed wound on his left leg. The patient was under the influence of alcohol when he was injured. The infection spread, causing peripheral arterial disease, which eventually led to the development of gangrene till mid-calf of the left leg, for which he was amputated. Above-knee amputation with a fish-mouth incision was performed. Physiotherapy management focused on prehension and grip exercises for right upper limb, strengthening of all the limb musculature, stump management, transfer training, gait training with walker/crutches and prosthesis, and home exercise program, retraining activities of daily living. This case study embodies a rehabilitation program for this patient who suffered from stroke followed by amputation of the left leg. It focuses on bringing the patient back to his near-normal life. Regular physiotherapy helped the patient build up confidence, helped in resolving his addictions, and provided individual structure management. We present a rare case of above-knee amputation secondary to complications of alcohol abuse where an extensive post-op care and elaborate physiotherapy program resulted in a successful recovery.

## Introduction

Amputation is the removal or loss of all or part of an extremity like toes, arms, legs, fingers, etc. This may hamper a person's activities and change the experience of the task. Most amputations are done to treat problems like gangrene, peripheral vascular disease, tumours, infection, congenital deformities, etc. Vascular disease comprises more than 90% of the lower limb amputation [1,2]. Heavy drinkers experience slurred speech and unsteadiness and alcohol can have a fatal effect that results in ventricular fibrillation, respiratory failure, or inhalation of vomit [3]. Although rare, alcohol can cause atherosclerotic stenosis of the microvasculature. This can involve a variety of vasculature-causing coronary artery disease, cerebrovascular diseases, and/or peripheral vascular diseases [4].

There are three compartments around the femur. Quadriceps and genu articularis correspond to the anterior compartment, and the femoral nerve is also a part of this compartment. The proximal portion of the sartorius muscle comes under the anterior part, and the distal is under the medial part of the thigh. The medial compartment contains the adductors and gracilis, which are supplied by the obturator nerve. Only the adductor magnus muscle is innervated by the sciatic nerve; sensory supply is by the saphenous nerve. Branches of the sciatic nerve, semitendinosus, short and long head of biceps brachii, and semimembranosus are all part of the posterior portion of the thigh [5].

The level of amputation is decided according to the course of the disease. When a disease involves the region of thigh or knee, above knee amputation is performed via the femur bone and thigh musculature, and the muscles of the thigh are sutured to the end of the bone to preserve the function of the following muscles: adductor magnus, rectus femoris, vastus lateralis, sartorius, long head of biceps femoris, semitendinosus, gracilis, tensor fasciae latae, short head of biceps femoris, and semimembranosus [6]. A number of complications can develop in patients not adherent to postoperative care. These include atrophy of muscle, contracture, scar adhesion, suture infection, wear and tear of the skin due to friction from prosthesis, phantom limb syndrome, and neuromas among others. In severe cases, they may develop psychological problems like depression, anxiety, panic disorder, substance abuse, etc. In addition, those with primary alcohol abuse may develop withdrawal syndrome, or relapse of alcohol abuse. These complications can be prevented with elaborate postoperative care [7]. Lack of knowledge on the use of prostheses makes the fitting procedure difficult for patients with above-knee amputation. This may further lead to the patient being borne in a wheelchair which can add up to more complications such as a decrease in capability to perform activities of daily living, bed sores, etc. which poses even more problems for elderly patients due to associated morbidities [8]. 

## Case presentation

A 65-year-old rickshaw driver presented to the hospital with extensive gangrene of the lower limb. He was a chronic alcoholic and a drug abuser (marijuana) for more than 40 years. The patient had an attack of stroke nine years back which affected his right side face and right upper limb. The patient claims to have stopped drugs for a year. The patient was apparently alright six months back, when he came home intoxicated with alcohol and marijuana, and had a splinter injury on the little toe of the left leg. He tried to remove the splinter with a razor blade but instead made small cuts with it on his toe. The wounds worsened over time and he developed gangrene of the little toe. He was taken to a private local hospital in Amravati where the gangrenous toe was removed and he was discharged on home medications. Unfortunately, blackening of the foot continued and gradually progressed upwards to the mid-calf of the left leg. This was associated with loss of sensation and loss of ability to move the toes and ankle joint. On examination, he had extensive dry gangrene of the left lower limb up to the knee with no pain, discharge, or bleeding. There were no sensations and absent peripheral pulses in the popliteal artery, anterior tibial artery, posterior tibial artery, and dorsalis pedis artery of the left lower limb, but normal findings on the right side. Tingling and numbness were present over the viable part of the left leg. On further work-up, he was diagnosed with thrombotic occlusion of the left iliac artery. After informed consent and medical optimization, he underwent an above-knee amputation. His post-operative course was unremarkable and he was discharged on home medication on the third day of admission along with a physiotherapy referral.

Clinical finding

We received the patient our department of physiotherapy on the third post-operative day . He was progressing well and had no active complaints. After an informed consent, his wounds were examined and found to have no discharge from the suture site, tenderness of grade 3 was present, pain on Numerical Pain Rating Scale was 7/10, and oedema was present in both upper extremities. Timeline of this patient is shown in Table 1.

**Table 1:** Timeline

Date of admission	07/09/2022
Date of surgery	13/09/2022
Date of physiotherapy referral	16/09/2022
Date of discharge	28/09/2022

Diagnostic assessment

**Figure 1:**
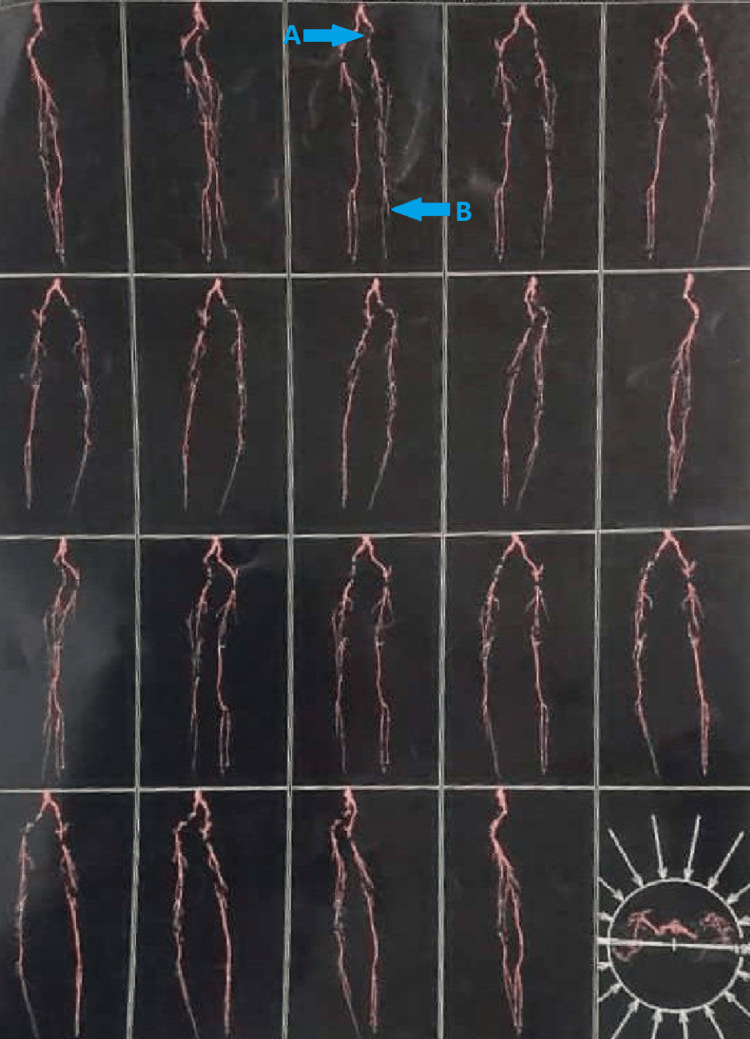
Arterial study of left lower extremity, done through multislice 3D CT angiography of both legs which shows impression (A) narrowing of artery and thrombus seen in left iliac artery, common femoral, superficial femoral artery, popliteal artery, and anterior and posterior tibial artery. For which patient was operated for toe amputation (B) absent flow of blood in left dorsalis pedis artery, first metatarsal artery and dorsal metatarsal arteries of left foot shown by the blue arrow. 3D- 3-dimensional. CT- computerized tomography.

The following investigation for this case was done 20 days before date of admission. A 3D angiography of both the legs are shown in Figure 1. It shows the arterial supply of both the lower extremities. 

Physiotherapy assessment 

**Figure 2:**
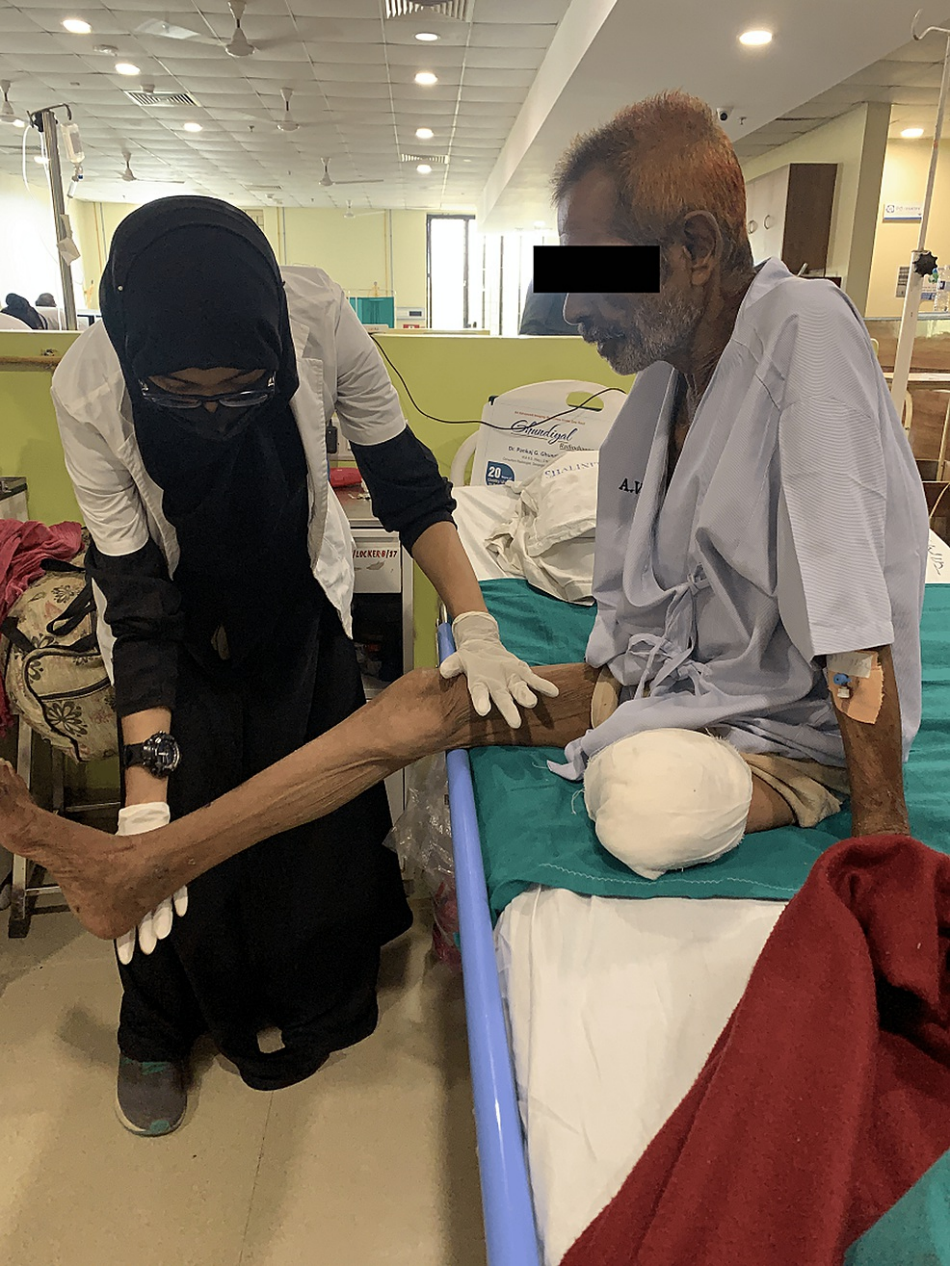
Assessment of strength of knee extensors.

Physical therapy assessment is one of the essential procedures to determine a patient's strength (Figure 2), mobility, the ability to do activities of daily living, functional independence, static and dynamic balance, gait, etc. For this patient, assessment focused on the strength of upper limb and lower limb and range of motion of appendicular joints. The assessment was done on the third post-operative day and the progression was checked after postoperative physiotherapy protocol was completed. Table 2 shows pre-physiotherapy assessment. Table 3 shows post-physiotherapy assessment values.

**Table 2:** Pre-physiotherapy assessment MMT- Manual Muscle Testing ROM- Range of Motion MCP- Metacarpal phalanx PIP- Proximal interphalanx DIP- Distal interphalanx

MMT		Right	Left
Shoulder	Flexors	4/5	+4/5
	Abductors	4/5	+4/5
Elbow	Flexors	-4/5	+4/5
	Extensors	-4/5	+4/5
Wrist	Flexors	3/5	+4/5
	Extensors	+3/5	+4/5
Finger	Flexors	-3/5	+4/5
	Extensors	-3/5	+4/5
Hip	Flexors	+3/5	-3/5
	Abductors	+3/5	-3/5
Knee	Flexors	+3/5	-
	Extensors	+3/5	-
Ankle	Planterflexors	4/5	-
	Dorsiflexors	4/5	-

ROM		Right		Left	
		Active	Passive	Active	Passive
Shoulder	Flexion	0˚-170˚	0˚ -177˚	0˚-175˚	0˚-180˚
	Abduction	0˚-169˚	0˚-180˚	0˚-175˚	0˚-180˚
Elbow	Flexion	0˚ -150˚	0˚-160˚	0˚-157˚	0˚-160˚
	Extension	-	-	-	-
Wrist	Flexion	0˚-50˚	0˚-60˚	0˚-90˚	0˚-100˚
	Extension	0˚-30˚	0˚-35˚	0˚-60˚	0˚-65˚
MCP	Flexion	0˚-30˚	0˚-32˚	0˚-90˚	0˚-110˚
	Extension	0˚-10˚	0˚-13˚	0˚-40˚	0˚-45˚
PIP	Flexion	0˚-30˚	0˚-32˚	0˚-100˚	0˚-110˚
DIP	Flexion	0˚-20˚	0˚-21˚	0˚-78˚	0˚-80˚
Hip	Flexion	0˚-110˚	0˚-115˚	0˚-90˚	0˚-95˚
	Abduction	0˚-45˚	0˚-47˚	0˚-30˚	0˚-33˚
Knee	Flexion	25˚-130˚	25˚-134˚	-	-
	Extension	130˚-25˚	130˚-28˚	-	-

**Table 3:** Post-physiotherapy assessment MMT- Manual Muscle Testing ROM- Range of Motion MCP- Metacarpal phalanx PIP- Proximal interphalanx DIP- Distal interphalanx

MMT		Right	Left
Shoulder	Flexors	5/5	5/5
	Abductors	5/5	5/5
Elbow	Flexors	5/5	5/5
	Extensors	5/5	5/5
Wrist	Flexors	5/5	5/5
	Extensors	5/5	5/5
Finger	Flexors	5/5	5/5
	Extensors	5/5	5/5
Hip	Flexors	5/5	5/5
	Abductors	5/5	5/5
Knee	Flexors	5/5	-
	Extensors	5/5	-
Ankle	Planterflexors	5/5	-
	Dorsiflexors	5/5	-

ROM		Right		Left	
		Active	Passive	Active	Passive
Shoulder	Flexion	0˚-180˚	0˚-185˚	0˚-180˚	0˚-185˚
	Abduction	0˚-180˚	0˚-185˚	0˚-180˚	0˚-185˚
Elbow	Flexion	0˚-160˚	0˚-165˚	0˚-160˚	0˚-165˚
	Extension	-	-	-	-
Wrist	Flexion	0˚-90˚	0˚-110˚	0˚-90˚	0˚-110˚
	Extension	0˚-70˚	0˚-85˚	0˚-70˚	0˚-85˚
MCP	Flexion	0˚-85˚	0˚-90˚	0˚-90˚	0˚-90˚
	Extension	0˚-45˚	0˚-47˚	0˚-45˚	0˚-47˚
PIP	Flexion	0˚-90˚	0˚-110˚	0˚-110˚	0˚-120˚
DIP	Flexion	0˚-80˚	0˚-85˚	0˚-80˚	0˚-85˚
Hip	Flexion	0˚-130˚	0˚-135˚	0˚-130˚	0˚-135˚
	Abduction	0˚-45˚	0˚-49˚	0˚-45˚	0˚-50˚
Knee	Flexion	25˚-134˚	25˚-134˚	-	-
	Extension	130˚-28˚	132˚-28˚	-	-

Physiotherapy rehabilitation 

**Figure 3:**
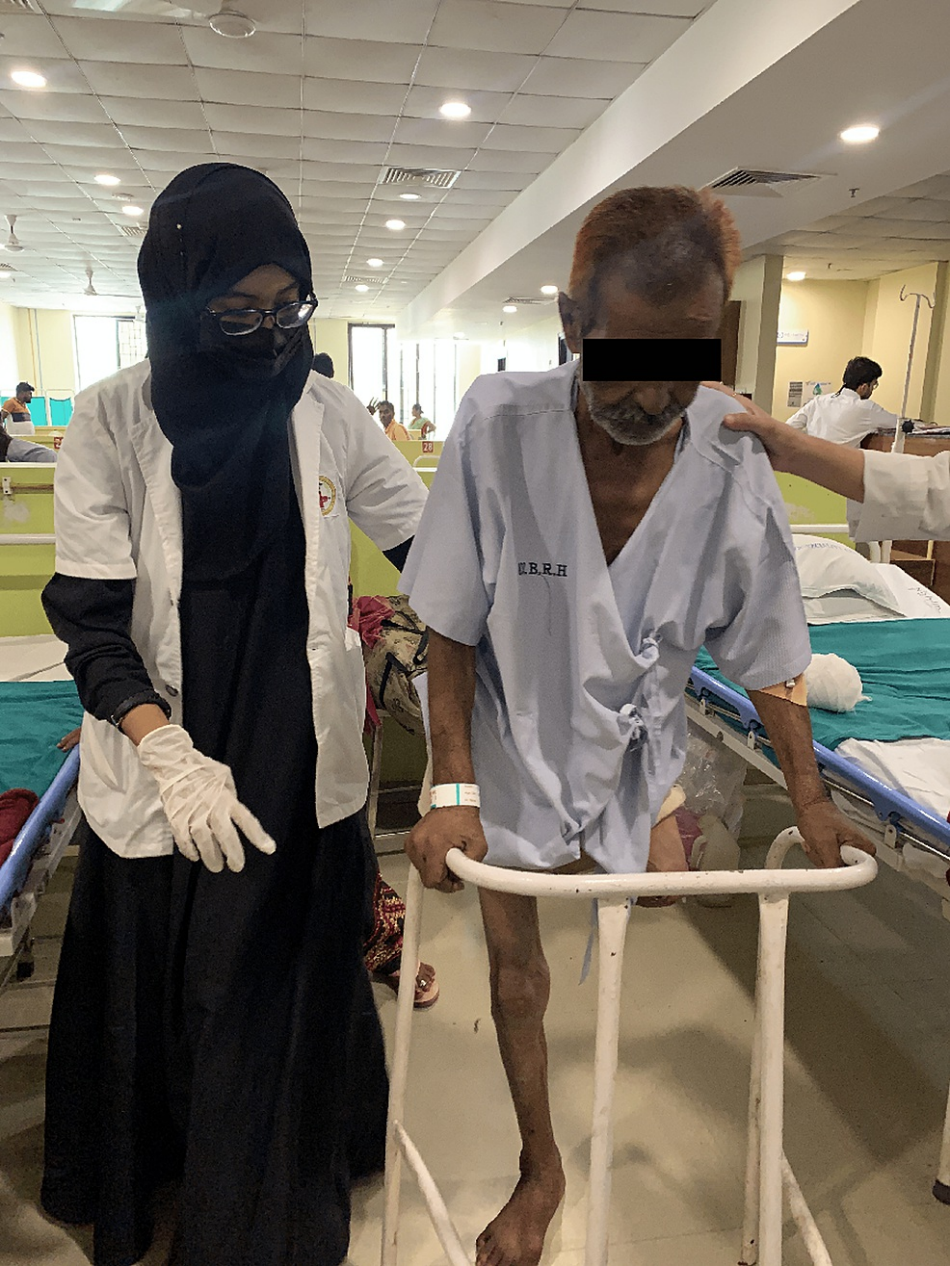
Gait training with walker.

One of the significant roles in the multidisciplinary care for post-amputees is individualized physiotherapy protocol. Physiotherapy rehabilitation is divided into preoperative and postoperative regimens. Preoperative physiotherapy management consists primarily of patient education and training the patient for postoperative rehabilitation, like strengthening of upper limbs for the use of crutches after amputation, dynamic quadriceps etc. Postoperative rehabilitation is divided into three phases: acute, pre-prosthetic, and prosthetic. The patient is trained to become functionally independent, either with walker (Figure 3) or a prosthesis. Table 4 and Table 5 show preoperative and postoperative physiotherapy rehabilitation, respectively.

**Table 4:** Preoperative physiotherapy rehabilitation reps- repetitions

Preoperative	Goals	Intervention	Treatment dose
	To give awareness of the condition to the patient and family.	Educate the patient and caregivers about the importance of physiotherapy. Counseling for control of addiction. Psychological counseling.	
	To prevent circulatory obstruction.	1. Ankle pumps. 2. Dynamic quadriceps.	10 reps × 3 sets
	To prevent respiratory complications.	1. Thoracic expansion 2. Pursed lip breathing.	10 reps × 3 sets
	To stretch, and Strengthen individual muscles of the upper and lower limbs. To prevent extensor lag.	1. Stretching for the right hand. 2. Weight lifting according to 1RM for shoulder, elbow, wrist, and fingers. 3. Vastus medialis strengthening.	10 reps × 3 sets
	To train gait with walker/crutches.	1. Focus on each component of gait cycle. Explain the different gait patterns.	100 feet × 3 sets
	To teach techniques for suture care.	1. Prevent friction. 2. Prevent infection.	

**Table 5:** Postoperative physiotherapy rehabilitation reps- repetitions

Postoperative			
Acute phase	Stump management and pain management.	1. Elevate limb. 2. Bandaging. 3. Positioning in bed/wheelchair.	10 reps × 5 sets
	Prevent secondary complications.	1. Ankle pumps. 2. Gluteal sets. 3. Dynamic quadriceps.	10 reps × 5 sets
	Transfer training.	1. Bed mobility. 2. Training to get in and out of the toilet/bed/wheelchair.	10 reps × 5 sets
Pre-prosthetic phase	Strengthening.	1. Strengthening of limbs along with range of motion exercises.	10 reps × 5 sets
	Stretching.	1. Stretching of lower limbs.	10 reps × 5 sets
	To advise for prosthesis and teach prosthetic care.	1. Bandaging techniques. 2. Fitting of the prosthesis.	
Prosthetic phase	Gait training with prosthesis.	1. Gait training with walker/crutches. 2. Retraining gait variables. 3. Walking on different terrains.	Progressively increase the distance starting from 100 feet for 3 sets.
	Home ex program	1. Strengthening. 2. Avoid one position for long duration. 3. Stretching. 4. Ergonomic advice.	10 reps × 3 sets

## Discussion

Chronic alcoholism leads to insufficiency in the blood supply, especially the peripheral nerve, and if an infection occurs due to an injury it may lead to gangrene of the lower extremities. No definite therapeutical intervention can treat gangrene except for amputation of that part as stated in an article by Camargo et al. [9]. A review article published in 2014 by Mckechnie et al. showed the prevalence of psychological problems in patients with traumatic amputation, which affected their relationship with their family members and colleagues, causing social withdrawal and affecting their quality of life [10]. According to an article published by Benveniste et al. in 2022, a delay in wound healing occurs in chronic alcoholics and this possesses a great risk of infection if not taken care of properly; they are also prone to more risk of injury. This article demonstrated the healing process in alcoholic mice and control mice. Poor healing took place in alcoholic mice; this delay occurred due to the failure of cells to migrate to polyvinyl sponges which were implanted in mice containing 10% ethanol and showed less deposition of collagen in the early stage but no inhibition of tissue repair [11].

An analysis done by Piano et al. showed that drinking alcohol on a long-term basis not only predisposes to hypertension but also causes coronary heart disease, stroke, peripheral arterial disease, altered platelet response, and cell apoptosis [12]. Marijuana, with its wide range of effects on cardiovascular events, has also been shown to cause vasoconstriction of peripheral blood supply, causing gangrene according to a study performed by Latif et al. [13]. For a transtibial amputation, the correlation between a number of medical problems and different age groups on post-amputation mobility was done, resulting in a negative relationship between age and post-amputation mobility, meaning poor mobility in elderly patients, as found in a research article from 1995 written by Johnson et al. [14].

## Conclusions

Post-amputation rehabilitation consists of three phases: acute, pre-prosthetic, and prosthetic phases. This protocol helped in gaining back strength of the affected limb and training gait pattern with both prosthesis and walker/crutches. Early physiotherapy rehabilitation plays an important role in preventing complications occurring after amputation. With the basic strengthening and retraining protocol, the patient showed a very good outcome and restored the quality of life and improved functional independence.
